# Biosynthesis of selenium nanoparticles and their protective, antioxidative effects in streptozotocin induced diabetic rats

**DOI:** 10.1080/14686996.2020.1788907

**Published:** 2020-07-27

**Authors:** Dabei Fan, Li Li, Zhizhen Li, Ying Zhang, Xiaojun Ma, Lina Wu, Haohao Zhang, Feng Guo

**Affiliations:** a Division of Endocrinology, Department of Internal Medicine, The First Affiliated Hospital of Zhengzhou University, Zhengzhou, China; b Ophthalmologic Center, The First Affiliated Hospital of Zhengzhou University, Zhengzhou, China

**Keywords:** Se NPs, H_2_SeO_3_, streptozotocin, insulin, 100 Materials

## Abstract

Green synthesis of selenium nanoparticles (Se NPs) was performed by mixing *Hibiscus sabdariffa* (*roselle plant*) leaf extract with the solution of selenious acid (H_2_SeO_3_) under continuous stirring conditions resulting the *roselle plant* secondary metabolites conjugated Se NPs. The existence of functional groups of *roselle plant* secondary metabolites on the surface of prepared Se NPs was confirmed by Fourier transform infrared spectroscopy (FTIR). The formation of crystalline nanoparticles with anisotropic shape was confirmed by transmission electron microscopy (TEM) images. Furthermore, we also studied anti-oxidative and protective effects of Se NPs in streptozotocin (STZ) induced diabetes rats. These STZ induced diabetic rats were daily exposed to Se NPs or/and insulin treatment and the effect of Se NPs on the factors correlated to oxidative damage in the rat testes were evaluated. The biochemical studies showed that the Se NPs are capable to enhance the serum testosterone reduction caused due to STZ induced diabetes. In addition, Se NPs can significantly reduce the oxidative stress indicators of the testicular tissue such as nitric oxide and lipid peroxidation. However, the treatment of Se NPs on the STZ induced diabetic rats increased the activities of antioxidant enzyme as well as the glutathione content in testicular tissues. Furthermore, microscopic studies revealed that the Se NPs are capable of preventing the histological damage in the testes of STZ induced diabetic rats. Altogether, these results explained the possible effects of Se NPs in attenuating oxidative damage induced by diabetes, especially in the testicular tissue.

## Graphical abstract

**Figure:**
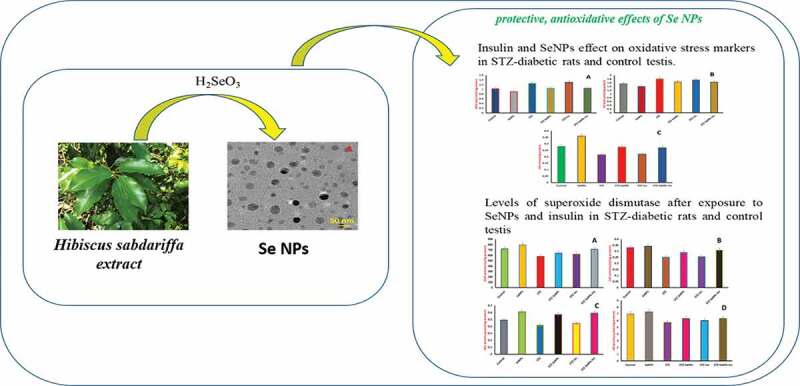

## 1. Introduction

Selenium (Se) is one among the trace elements necessary for humans. In human body, selenium is involved in various processes together with antioxidant defense and immune functions. However, the deficiency of selenium may cause osseous, cardiac, immune and muscular disturbances in the human body [1,2]. The existence of selenocysteine amino acid in the proteins is mainly responsible for biological functions of selenium. Earlier studies have reported that around 100 selenoproteins were found in mammals [2]. Among them, antioxidant enzymes such as thioredoxin reductase and glutathione peroxidase along with selenoprotein-P, are responsible for the transport and storage of Se [3–6]. In addition, it was already known that selenium supplements are protective against wide varieties of harmful factors involving physical factors, such as magnetic fields or heat stress and chemical factors, like drugs causing critical side effects, carcinogens, pesticides or mycotoxins and heavy metals. However, it is a complex issue to consider Se as a most effective supplement because of its narrow therapeutic index, applied form and its efficacy that depends on the method and dosage used in the treatment [5,7–10].

The median lethal dose of sodium selenite (Se of 7 mg/kg body weight) estimated in animal models is almost 20 times lower than that of sulfides of selenium, and it is more than 900 times lower than that of elemental Se [11]. As per the US National Academy of Sciences, the daily intake of Se recommended is 55 µg for adults; however, it should not exceed the threshold limit of 400 µg [7]. On the other hand, a dose greater than 700 µg/day is considered to be toxic for adults as per earlier reports [12]. Selenium toxicity shows symptoms such as fatigue, disturbances in nervous, cardiovascular, respiratory and gastrointestinal systems as well as in connective tissues [11,13]. The attention of researchers in selenium and its effects on human health is increasing day-by-day and a wide range of selenium compounds which include organic, inorganic and natural products enriched with Se such as green tea probiotics, yeast and Se NPs were studied [3,8,10,14–18]. In recent times, organic compounds are widely studied as some of them show similar activity (e.g., diphenyl diselenide or ebselen) to that of glutathione peroxidase [19]. Furthermore, it was proved that diphenyl diselenide also possess many pharmacologically beneficial properties like hepatoprotective, anti-hyperlipidemic, antidepressant, anti-hyperglycemic and antiulcer effects [19,20].

Therefore, the present study was intended to use *roselle plant* leaf extract as reducing agent for the green synthesis of Se NPs. *Hibiscus sabdariff*, also recognized as roselle, which is a supreme crop for emerging countries as it is comparatively easy to produce. The seed oil and other plant materials have important medicinal values. The leaves of *roselle plant* mainly constitute of ascorbic acid, proteins and carbohydrates which play a key role in the synthesis and formation of Se NPs [21]. It is well reported that plant extracts were already utilized in the synthesis of various nanomaterials [22–27]. Also, the prepared Se NPs were studied for their important effect to ameliorate the testicular dysfunctions in diabetic rats, by specifically reducing oxidative stress.

## 2. Materials and methods

### 2.1. Preparation of roselle plant leaf extract

Leaves collected from *roselle plant* were washed using sterile distilled water in order to eliminate dust particles and then dried under shade. The leaf extract preparation was carried out by adding 10 g of finely cut dried leaves of *roselle plant* into a 500 mL beaker together with deionized water of 500 mL. On heating for 15 min at 70°C, the aqueous solution turned from colorless to yellow. Later, the resultant mixture was cooled at room temperature and then filtered using Whatman no.1 filter paper. The obtained mixture was then subjected to centrifugation for the removal of biomolecules at 1500 rpm for about 5 min. The obtained supernatant extract was stored at a temperature of 25°C for future use.

### 2.2. Synthesis of Se NPs

Selenious acid of 50 mM concentration was added to the plant extract of 100 mL and then stirred for 5 min. Then, the obtained solution was subjected to incubation at a temperature of 20–22°C. Change in color of reaction mixture was observed and analyzed by UV-Vis spectrophotometer. After completion of the reaction, the reaction mixture was centrifuged at 1500 rpm to obtain Se NPs. The Se NPs were then purified with the help of acetone and distilled water followed by and drying overnight. Furthermore, the Se NPs were ultrasonically suspended in PBS (pH 7.4) followed by centrifugation. The obtained red colored Se NPs were utilized for further analysis.

### 2.3. Animals

Adult Wistar rats (male) of about 110 to 140 g weight and 2 months old were utilized for the current study. All the animals were provided with free access to food and water. All studies on animals were carried out in accordance with the animal care standards and all experiments were performed according to institutional animal ethical committee guidelines. Diabetes was induced in rats upon injecting streptozotocin (STZ; Sigma, Shanghai) intraperitoneally (i.p.) with a dose of 55 mg/kg body weight. The fresh solution of STZ was prepared immediately prior to injection, by diluting in 0.05 M concentration of citrate buffer of pH 4.5. The blood drops were collected from the STZ induced diabetic rats for every 3 days in order to monitor the blood glucose levels of the rats and the blood drops were tested using an Accu-check blood glucose meter. STZ induced diabetic rats with glucose levels in the blood greater than or equal to 200 µg/dl (15 mM) after 7 days of STZ injection, were considered as hyperglycemic. On the other hand, only citrate buffer was injected on the same type of rats in the control group.

### 2.4. Experimental design

STZ induced diabetic rats were separated in four groups with 7 rats into every group. Control group animals, which were injected only with citrate buffer, were separated into two groups with seven rats in each.

The first animal group of STZ induced diabetic rats (STZ group) was administrated with the solution of physiological sodium chloride. In the second animal group, STZ induced diabetic rats were administrated with Se NPs of dose 0.1 mg/kg body weight, once in a day (STZ-Se NPs group). The third animal group of STZ induced diabetic rats were daily treated with 6 U of insulin per kg body weight subcutaneously (STZ-Ins). In the fourth animal group, STZ induced diabetic rats were administrated with both insulin and Se NPs in above-mentioned concentrations (STZ-Ins-Se NPs). In addition, other two control groups having 14 rats (citrate buffer injected) were injected with any one of the sodium chloride solution (control group) or Se NPs of dose 0.1 mg/kg (Se NPs group). Although insulin was given subcutaneously every day for 28 days and all treatments of Se NPs and buffer were orally administrated [28]. In addition, a syringe puncture was utilized to isolate rat serum from the blood that was collected in the abdominal aorta of rat. This serum was further used for histological studies, biochemical studies and/or molecular studies. The weights of all the rats were recorded before performing various treatments, and again before sacrificing.

### 2.5. Testes index changes in rats

The relative weight of the testes was determined as follows by using the weight of the left testis (LT),

(Left testis weight/body weight)X100.

### 2.6. Serum testosterone estimation

The quantitative estimation of serum testosterone from rats was performed with the help of a technique called enzyme-linked immunosorbent assay by using a kit that was specific to quantify serum testosterone in blood sample collected from rats. This study was carried out according to the instructions given by the manufacturer.

### 2.7. Preparation of testis homogenates

Ten volumes of 50 mM concentration tris hydrochloride ice cold medium with a 7.4 pH were used to homogenize the testes. Homogenized testes were subjected for centrifugation at 1000 × g at 4°C for 10 min. Moreover, the supernatants were utilized to investigate the activities of antioxidant enzyme and oxidative stress. One of the early reported methods [29] was used to measure the protein level in the homogenate.

### 2.8. Oxidative stress of testes

The testes homogenate was subjected to incubation with 1 mL of 0.67% thiobarbituric acid and 1 mL of 10% trichloroacetic acid (TCA) at 100°C for half an hour to evaluate the lipid peroxidation (LPO) values in the testes [30]. A colored complex was formed when malondialdehyde (MDA) reacts with TCA, and expresses the quantity of MDA with a maximum absorbance at 535 nm. In the meantime, optimized acid reduction method was utilized to determine the level of nitric oxide (NO) in the testes homogenate as reported in one of the earlier reports [31]. Moreover, the testicular glutathione (GSH) was evaluated through the reduction of Elman reagent as reported earlier [32].

### 2.9. Antioxidant status

The activities of various antioxidant enzymes have been identified as indicators for assessing oxidant stress in the testes. As described earlier, superoxide dismutase (SOD) was evaluated depending on the ability of SOD to inhibit the nitroblue tetrazolium (NBT) [33]. Later, the testicular catalase (CAT) activity that was necessary for the removal of H_2_O_2_ formed by the superoxide dismutase was determined by mixing testis homogenate of 50 µL to H_2_O_2_ of 30 mM concentration in a 50 mM concentration of potassium phosphate buffer with 7.8 pH. The H_2_O_2_ consumption was quantified for 120 sec at time intervals of 20 sec Sizer and Beers by using photometer at wavelength of 340 nm [34]. The expression of testicular catalase activity and superoxide dismutase activity was represented as units/mg proteins. Furthermore, glutathione reductase (GR) was indirectly assayed depending on the oxidative reaction NADPH to NADP^+^. The decrease in NADPH absorbance at 340 nm expressed a reduction of H_2_O_2_ to alcohol by glutathione peroxidase (GPx). Similarly, the indirect examination of GPx activity in the testis homogenate was determined by performing an assay described in an earlier report [35]. In the current assay, the excess GR regulates the oxidized glutathione (GSSG) produced from GPx activity. The GR activity was observed by following the loss of NADPH at 340 nm.

### 2.10. Histological changes

Neutral-buffered formalin (10%) was used to fix the testis tissues for a day followed by dehydrating using ethyl alcohol. Later, xylene solution was used to clean the tissues that were fixed and placed on molten paraplast. The range of tissue sections were managed to be 4–5 µm. Furthermore, the tissue sections were stained using haematoxylin-eosin to visualize the sperm cells and seminiferous tubules using a Nikon microscope.

### 2.11. Characterization

Lambda-19, PerkinElmer UV-Vis spectrophotometer, Japan was utilized to characterize the formed Se NPs in order to understand their kinetic behavior. Sample measurements were recorded at the scanning range of 200–800 nm with a scan speed of 480 nm/min. An aqueous colloidal dispersion of Se NPs was used for optical absorption measurements. Furthermore, to prepare translucent sample discs, Se NPs of 2 mg were encapsulated in KBr pellet of 100 mg. Fourier transform infrared (FTIR) spectroscopy was performed with pelleted sample specimens using Bruker IR Affinity instrument, Japan, in the wavenumber range of 400 to 4000 cm ^−1^ with 1 cm^−1^ resolution. X-ray diffraction analysis was performed using X′Pert Pro PANalytical, ALMELO, Netherlands, in order to identify the structural characterization of the formed Se NPs. XRD analysis was performed at an operating voltage of 45 kV and 40 mA current in the 2θ range of 0° to 80°. A JEOL JSM 1200EX-II TEM microscope, Japan was used to record the transmission electron micrographs. A diluted dispersion of Se NPs was drop casted onto the surface of copper grid and then dried for TEM observation. Additionally, the same sample of Se NPs dispersion was used for size distribution analysis using a Horiba Scientific Nanoparticci (SZ-100), Japan instrument.

### 2.12. Statistical analysis

Data were expressed as mean ± standard error of the mean (SEM). Values were statistically studied using one-way analysis of variance (ANOVA). Duncan’s test was utilized as a post hoc to compare significance among groups as per the SPSS version 20.0. P value < 0.05 was considered as statistically significant.

## 3. Results and discussion

### 3.1. Se NPs characterization

**Figure 1.**
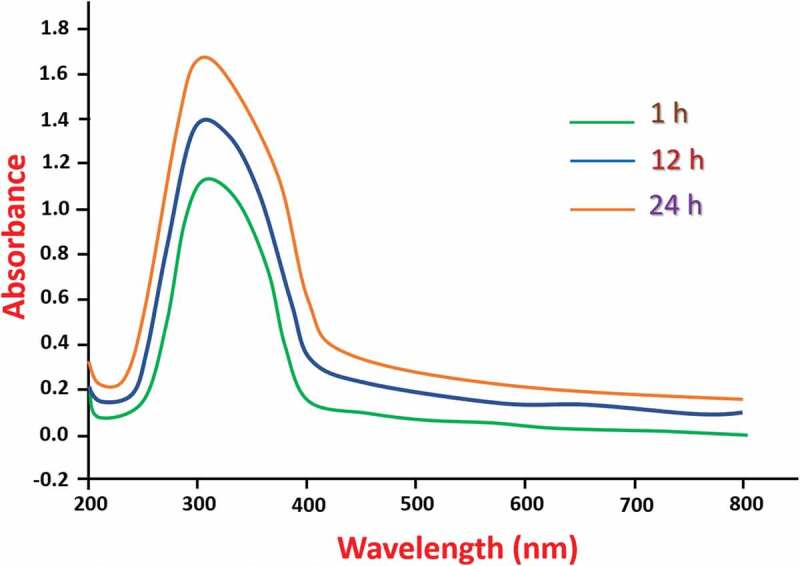
Evolution of optical absorption signal during the synthesis of Se NPs.

The synthesis of Se NPs is examined by UV-Vis spectroscopy by slow elevation in the absorbance wavelength at 320 nm with respect to the reaction time (Figure 1). The formation of Se NPs is identified visually by the change in color of reaction mixture from transparent to ruby red with time. No further changes in the color of reaction mixture were noticed after a day of incubation. The successful formation of Se NPs using the biomolecules present in the leaves of *roselle plant* is revealed by this observation. The absorption maximum (λ_max_) of Se NPs recorded at a wavelength of 320 nm after 1 day by UV-Vis spectroscopy (Figure 1), attributed to the surface plasmon resonance (SPR) of Se NPs. It is reported that the SPR of Se NPs causes a maximum absorption between 200 nm to 400 nm in the optical absorption spectrum [36].

**Figure 2.**
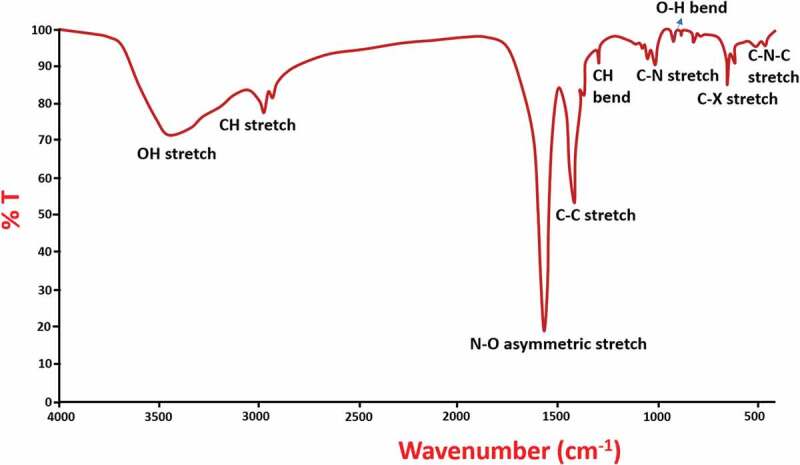
FTIR spectra of prepared Se NPs.

Figure 2 shows the results of FTIR analysis for the synthesized Se NPs. The presence of a broadband at 3434 cm^−1^ is attributed to the O-H group stretching of phenols and alcohols. An absorption band present at 2977 cm^−1^ and 2938 cm^−1^ corresponding to the C-H group stretching of alkynes. Also, the asymmetric stretch of N-O group of nitro compounds causes a band at 1571 cm^−1^. Due to the stretching of C-C group (in ring) in aromatics, a strong band is formed in FTIR spectrum at 1426 cm^−1^. The presence of a sharp peak at 1375 cm^−1^ attributed to the bending of C-H group in alkanes and the bands present at 1012 cm^−1^, 1077 cm^−1^ and 1044 cm^−1^ corresponds to the stretching vibrations of C-N group in amines. The bending vibrations of O-H groups in carboxylic acids cause a band at 923 cm^−1^. Similarly, the bands at 649 cm^−1^ and 817 cm^−1^ are due to stretching vibrations of C-X groups in alkyl halides and the bending vibrations of C-N-C group in amines cause bands at 461 cm^−1^ and 510 cm^−1^. According to these results, the stabilization and reduction of the Se NPs occur with the involvement of different functional groups of biomolecules found in plant extract. It is already reported that the phytochemicals act as stabilizing agents in the fabrication of metal nanoparticles [37]. However, further validation is necessary for the identification of active molecules which were responsible for Se NPs synthesis.

**Figure 3.**
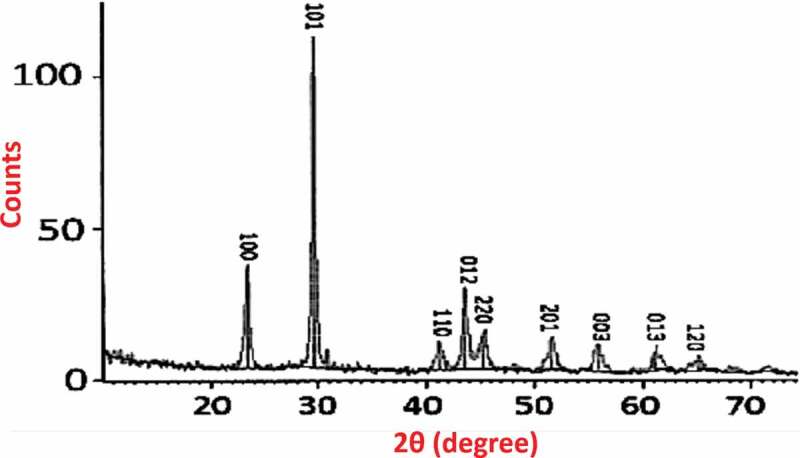
(a) XRD pattern of Se NPs with the stick pattern of JCPDS card 06–362.

Figure 3 shows the XRD pattern of prepared Se NPs. It reveals the crystalline nature of the formed Se NPs, which is indexed in accordance with the JCPDS file no. 06–362 [38–40]. The Scherrer’s equation is used to calculate the crystallite size of the prepared Se NPs as 35 nm.

**Figure 4.**
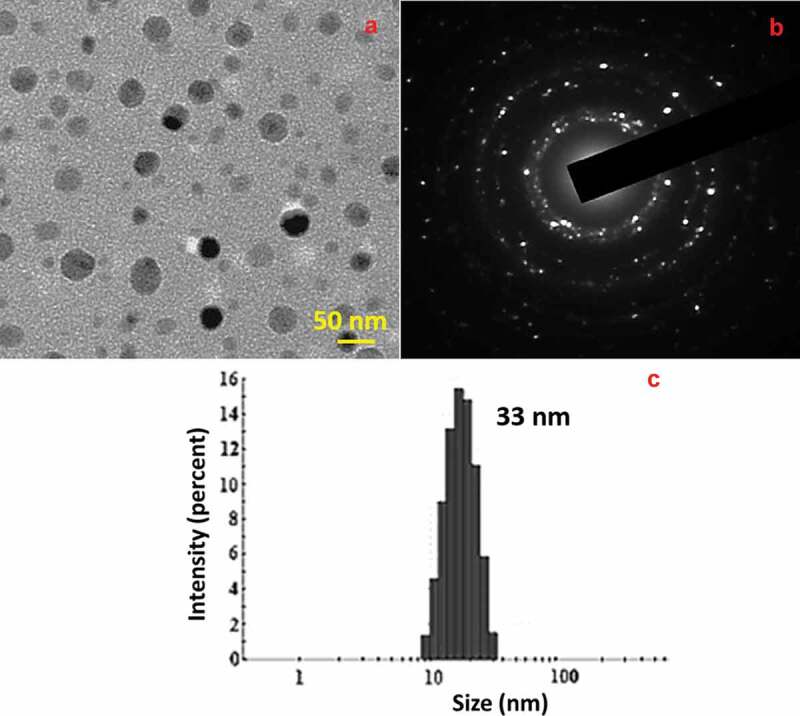
TEM image (a) SAED pattern (b) and DLS histogram (c) of prepared Se NPs.

Transmission electron microscopy (TEM) images and selected-area electron diffraction (SAED) patterns infer the crystalline nature of Se NPs with anisotropic shape including spherical, triangular and hexagonal particles (Figure 4(a)) [41]. Also, the SAED pattern of Se NPs shown in Figure 4(b) indicated the crystalline nature of the formed NPs with the existence of bright diffraction spots. Further, the DLS size distribution analysis presented in Figure 4(c) shows the presence of 20–50 nm sized particles with an average particle size of 33 nm, which is also in consistent with TEM results.

**Figure 5.**
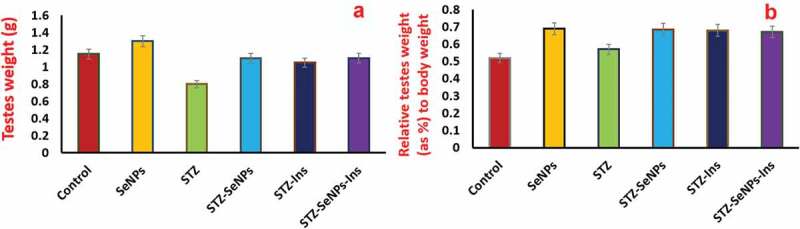
Effect of Se NPs and/or insulin on absolute weight of testes (a) and comparative weight of testes (b) of control and STZ induced diabetic rats. p < 0.05 is considered as significant.

Though STZ induced diabetic rats displayed a reduced weight of testes significantly when compared with rats in control group (), the testes weight relative to the total weight of STZ induced diabetic rats is unaffected (Figure 5(b)), which is because of the general weight loss in STZ induced diabetic rats. Surprisingly, treatment of insulin and/or Se NPs for 1 month has a significant effect (p less than 0.05) in the prevention of weight reduction in testes and in fact, the relative weight of testes is increased when compared with diabetic rats. Both the relative and absolute weight of testes are increased significantly by Se NPs in comparison with that of rats in control group. Hence, STZ decreased the body weight of rats including testes, whereas insulin and Se NPs increased the testes weight in rats, even in rats affected with diabetes.

**Figure 6.**
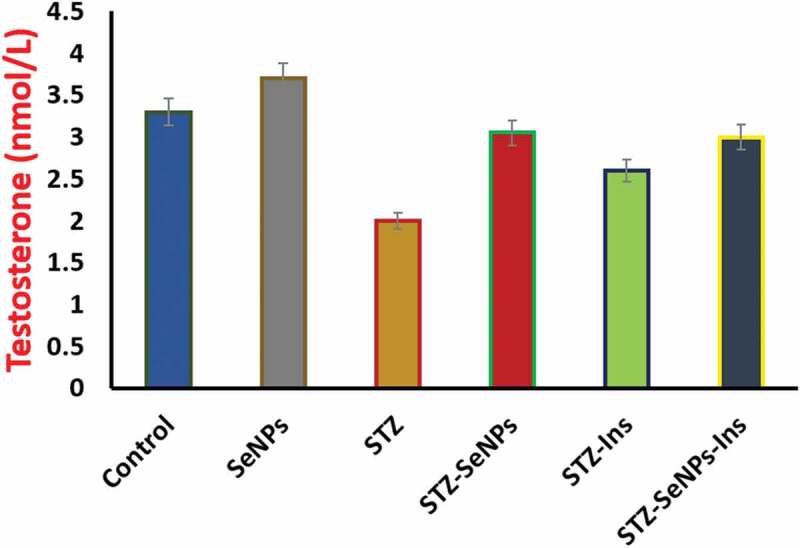
Effect of Se NPs and/or insulin on serum testosterone levels in control and STZ- diabetic rats. p < 0.05 is considered as significant.

A significant reduction (p less than 0.05) in the serum levels of testosterone is observed in diabetic rats when compared with the rats of control group. Surprisingly, non-diabetic rats showed an insignificant effect of Se NPs on increased levels of serum testosterone, indicating the role of Se NPs in the synthesis of testosterone (Figure 6). Moreover, the levels of serum testosterone are expected to recover significantly to normal levels by the administration of insulin or/and Se NPs for 1 month in non-diabetic rats (Figure 6).

**Figure 7.**
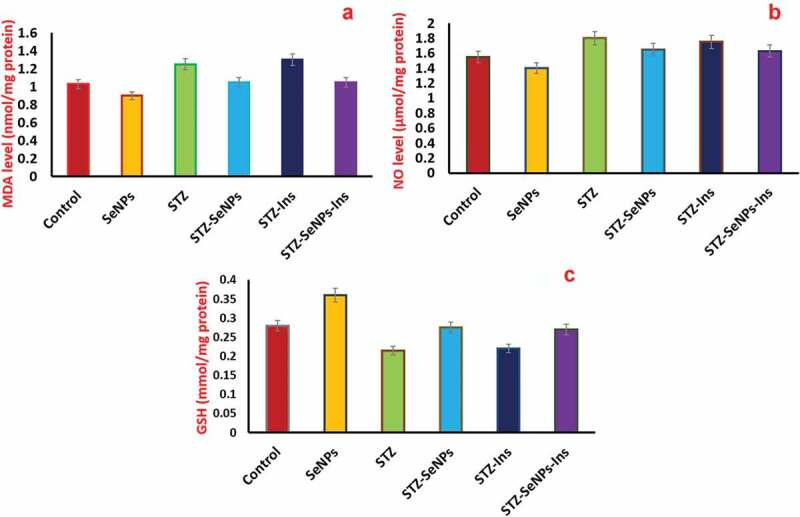
Effect of insulin and Se NPs on oxidative stress markers (a) peroxidation of lipids (b) NO levels (c) glutathione levels in testes of STZ-diabetic rats and control. p < 0.05 is considered as significant.

The STZ induced diabetic rats represented an increase in the nitric oxide (NO) and malondialdehyde (MDA) levels, when compared with the animals in control group (Figure 7(a,b)). Therefore, STZ caused an increase in the levels of NO and MDA, however, Se NPs administration resulted in decrease of their levels. Surprisingly, in STZ-induced diabetic rats and Se NPs treated animals showed the closed or similar levels of these markers (NO and MDA) to that found in control group animals (Figure 7(a), B, Se NPs compared with control and STZ). Treatment with insulin failed in the prevention of oxidative stress in testes with results that were similar to those of untreated diabetic rats (Figure 7(a), B, insulin compared to control and STZ). Moreover, the testicular tissue of rats induced with diabetes by STZ exhibited the significantly reduced levels of glutathione (GSH) (p less than 0.05) (Figure 7(c), STZ compared with control). Whereas, the treatment with insulin or/and Se NPs showed the significant attenuation of decreased levels of glutathione contents (Figure 7(c), insulin or/and Se NPs compared with STZ).

**Figure 8.**
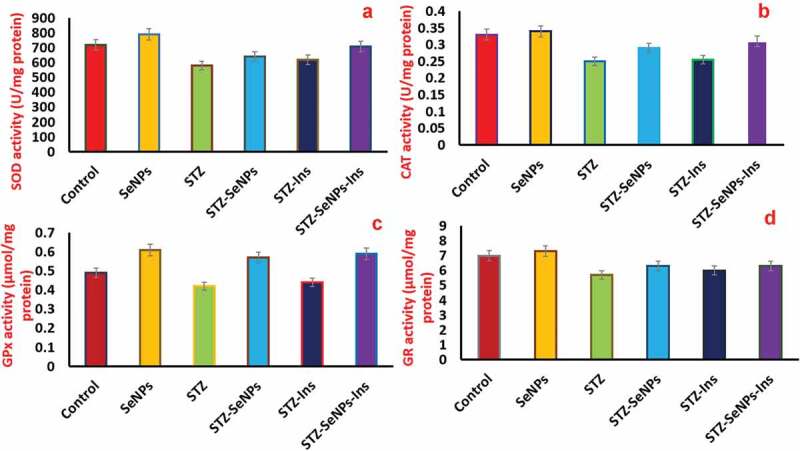
Effect of insulin and Se NPs on activity of antioxidant enzymes. Levels of superoxide dismutase (a) catalase (b) glutathione peroxidase (c) and glutathione reductase (d) after exposure to Se NPs, insulin in STZ-diabetic rats and control testis. p < 0.05 is considered as significant.

The function of antioxidants defense system was analyzed by measuring the enzyme activity of CAT, GR, SOD and GPx in order to study the diabetic effects with respect to oxidative damage to the testes. A significant decrease in the enzymatic activity of all enzymes was resulted by the induction of diabetes (Figure 8, comparison of STZ with the control group in all graphs). However, the activity of GPx is increased upon treatment with Se NPs in non-diabetic rats when compared with diabetic rats which were left untreated (Figure 8, comparison of Se NPs with control group in all graphs). Surprisingly, the activities of all the tested enzymes were enhanced with the treatment of Se NPs in STZ induced diabetic rats (Figure 8, comparing STZ-Se NPs with STZ in all graphs). Hence, the obtained results showed that the impaired activities of antioxidant enzymes in STZ-induced diabetic rats were recovered upon treatment with Se NPs.

**Figure 9.**
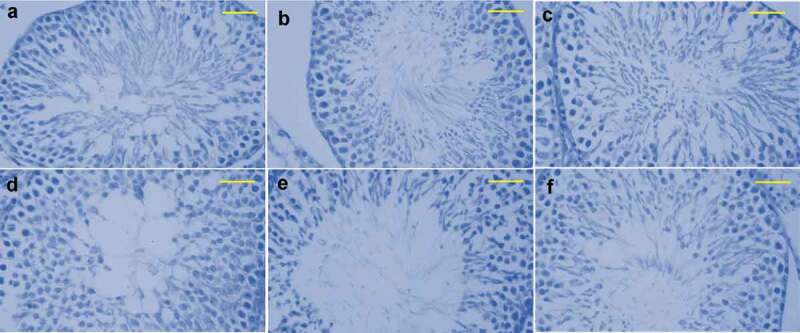
Histological images of the testis in rats. Spermatogenic cells obtained from testes of rats were undergone for specific antibody staining against PCNA. (a) control (b) Se NPs -treated rats (c) STZ-diabetic rats, (d–f) group induced with STZ and Se NPs, rats with insulin, STZ induction and rats induced with STZ- Insulin- Se NPs, correspondingly. Scale bar is 50 µm.

STZ-induced diabetic rats have shown that a high blood glucose level (≥298 mg/dL) has caused changes in the normal histological structure of the testes when compared with the control group rats (Figure 9, compare C with A). It is found that the epithelial layers were impaired severely, ranging from total to partial disorganization and also with definite impairment of organization in spermatogenesis stages. Furthermore, STZ-induced diabetic rats displayed atrophy in the seminiferous tubules with arrested necrotic and spermatogenesis cells visible in the luminal portion along with a lack of spermatogonia in the basal compartment, especially in the heavily damaged tubules. However, their normal structure was restored by the treatment with Se NPs (Figure 9 comparison of D with C). Histological staining of testis in STZ-diabetic rats exhibited a significant number of thickened seminiferous tubules that extended up to the basement membrane. Similar to control group rats, the diabetic rats which were treated with Se NPs regained their initial seminiferous tubules which were lined via series of spermatogenic cells layers (Figure 9(d)).

## 4. Conclusions

In conclusion, green synthesis of Se NPs using leaf extract of *roselle plant* was shown in the current study. The formation of crystalline nanoparticles with anisotropic shape was confirmed by the TEM analysis. In addition, the biochemical studies showed that the Se NPs are capable to enhance the serum testosterone reduction caused due to STZ induced diabetes. Furthermore, Se NPs can significantly reduce the oxidative stress indicators of the testicular tissue, namely nitric oxide and lipid peroxidation. However, the treatment of Se NPs on the STZ induced diabetic rats increased the activities of antioxidant enzyme as well as the glutathione content in testicular tissues. Additionally, the microscopic studies revealed that the Se NPs are capable of preventing histological damage occurring in the testes of STZ induced diabetic rats. Altogether, these results showed the possible effects of Se NPs in attenuating oxidative damage induced by diabetes, especially in the testicular tissue.
